# Factors associated with women’s autonomy regarding maternal and child health care utilization in Bale Zone: a community based cross-sectional study

**DOI:** 10.1186/1472-6874-14-79

**Published:** 2014-07-03

**Authors:** Dabere Nigatu, Abebe Gebremariam, Muluemebet Abera, Tesfaye Setegn, Kebede Deribe

**Affiliations:** 1 Department of Nursing, College of Medicine and Health Sciences, Madawalabu University, Bale-Goba, Ethiopia; 2 Department of Public Health, College of Medicine and Health Sciences, Bahir Dar University, Bahir Dar, Ethiopia; 3 Department of Population and Family Health, College of Public Health and Medical Sciences, Jimma University, Jimma, Ethiopia; 4 Brighton and Sussex Medical School, Falmer, Brighton, UK; 5 Addis Ababa University, School of Public Health, Addis Ababa, Ethiopia

**Keywords:** Women’s autonomy, Heath care utilization, MCH, Goba district, Ethiopia

## Abstract

**Background:**

Women's autonomy in health-care decision is a prerequisite for improvements in maternal and child health. Little is known about women’s autonomy and its influencing factors on maternal and child health care in Ethiopia. Therefore, this study was conducted to assess women’s autonomy and identify associated factors in Southeast Ethiopia.

**Method:**

A community based cross-sectional study was conducted from March 19^th^ until March 28^th^, 2011. A total of 706 women were selected using stratified sampling technique from rural and urban kebeles. The quantitative data were collected by interviewer administered questionnaire and analyzed using SPSS for window version 16.0. Descriptive statistics, bivariate and multiple logistic regression analyses were carried out to identify factors associated with women’s autonomy for health care utilization.

**Result:**

Out of 706 women less than half (41.4%) had higher autonomy regarding their own and their children’s health. In the multiple logistic regression model monthly household income >1000 ETB [adjusted odds ratio(AOR):3.32(95% C.I: 1.62-6.78)], having employed husband [AOR: 3.75 (95% C.I:1.24-11.32)], being in a nuclear family structure [AOR: 0.53(95% C.I: 0.33-0.87)], being in monogamous marriage [AOR: 3.18(95% C.I: 1.35-7.50)], being knowledgeable and having favorable attitude toward maternal and child health care services were independently associated with an increased odds of women’s autonomy.

**Conclusion:**

Socio-demographic and maternal factors (knowledge and attitude) were found to influence women’s autonomy. Interventions targeting women’s autonomy with regards to maternal and child health care should focus on addressing increasing awareness and priority should be given to women with a lower socioeconomic status.

## Background

The power balance between men and women plays an important role in the treatment seeking behavior of women. The power relationship that impedes women’s attainment of healthy and fulfilling lives operates at personal, society and to highly public levels [1]. Although the global estimates of women’s problems related to lack of autonomy are based on the agreed definition of women autonomy. The literature has defined it in different ways such as: “The ability to make decisions on one’s own, to control one’s own body, and to determine how resources will be used, without needing to consult with or ask permission from another person”. Women’s autonomy can be viewed as the control of women over their own lives, materials, access to knowledge and information, having equal say with their husbands or partners on matters affecting themselves and their families. It can also be equated with the authority to make independent decisions, freedom from constraint on physical mobility and the ability to forge equitable power relationships within families [2-5]. Furthermore, women’s autonomy is defined in three dimensions as: control over finance, decision-making power, and extent of freedom of movement [6]. Hence, women’s autonomy related to the extent of independent decision making, freedom from constraint on physical mobility and the ability to forge equitable power relationships within families, has been used for this study.

In many parts of Africa, women’s decision making power regarding reproduction and sexuality is extremely limited. Decisions on maternal health care are often made by husbands or other family members, which negatively influences maternal and child health service utilization [7]. A study done in Gambia showed that risk of child death is greater for women/primary caregivers with reduced financial autonomy compared to their counterparts. On the other hand a study done in Nigeria indicated that ethnicity was a determinant factor for wife's decision making authority [8,9].

Considering the making of large purchases as one indicator for women’s decision making autonomy, 42% and 44% of Ethiopian and Eritrean women respectively had no autonomy to make large purchases. The study also considered women’s attitude toward wife beating as indicator because women with high autonomy would not accept obvious gender power inequalities and any justification of husband to beat his wife. However, 83% of Ethiopian and 73% of Eritrean women believed that wife beating is justifiable for some occasional reasons [10].

Gender based power inequalities have been challenges to open communication between partners about reproductive health decisions and women's access to reproductive health care services which would contribute to poor health outcomes [1]. In Ethiopia, only 14.6% of married women decide autonomously on their health issues. Women have to wait for their husband’s decision on health care utilization for themselves and their children when the need arises. Poverty, distance to health care service, lack of education and awareness to use modern health care services, including reproductive health service, exacerbate the lowest level of autonomy [11].

The Ethiopian government has been striving to achieve Millennium Development Goal three (MDG-3), to promote gender equality and empower women, through designing and implementing policies and strategies giving emphasis on increasing women’s access to education and health care services. Furthermore, safeguarding women’s rights such as access to land, credit, and increasing the number of women benefiting from government programs and strategies, giving women leadership training have been the strategic turning points for women’s autonomy [12].

However, these programmatic level interventions especially on health care services have been based on inadequate systematic evidence that depicts the determinant factors of women’s autonomy regarding their own and their under-five children’s health care utilization which might be due to scarcity of data on women’s autonomy. Therefore, the objective of this study was to assess the level of women’s autonomy and predictors of women autonomy regarding their own and their under-five children’s health care utilization in the Goba District, Southeast Ethiopia.

## Methods

### Study setting and sample

A community based cross sectional study using quantitative methods of data collection was conducted in the Goba District in March 2011. The Goba district is one of the 18 districts in Bale Zone, Oromia Region of Ethiopia and located 444 km from Addis Ababa. The district has 24 rural and 4 urban kebeles with an estimated total population of 73,653 (including the Goba administrative town) of whom 37,427 were females; 32,916 (44.7%) of its population were urban dwellers [13]. A total of 43 health institutions were available in the district of which 13 were in urban areas of Goba district while 30 health facilities were found in the rural part of the district. The estimated total number of under-five children in the district (both rural and urban) is 12,153 [Goba Woreda Health Office: Annual report, unpublished]. All women who had under-five children in 12 randomly selected kebeles of Goba district were the source population.

The sample size was determined using a single population proportion formula considering 95% confidence level, 5% margin of error and 66% (p = 0.66) estimated proportion of married women who were able to decide on their own health matters with full or partial autonomy [14]. These figures were substituted in the formula bellow:

$$n_{i}=\frac{{\left(Z_{\frac{\alpha }{2}}\right)}^{2}p\left(1-p\right)}{d^{2}}=\frac{{\left(1.96\right)}^{2}\times 0.66\left(0.34\right)}{{\left(0.05\right)}^{2}}=344.8$$

**Where: n**_**i**_ is initial sample sizes, **Z**_**α/2**_ is critical value for normal distribution at 95% confidence level which equals to 1.96 (z -value at α =0.05), P is national level proportion of women who participate in decision making regarding their own health care (0.66) [14], **d** is a margin of error (0.05).

The calculated sample size, 345, was multiplied by a design effect of 2 and 10% of the calculated sample size, 69, was added for non-response. This made the final sample size 759. A total of 759 women who had under-five child from 12 kebeles (2 urban and 10 rural) were selected using stratified cluster sampling from both urban and rural settings. Preliminary household enumeration (census) was done to identify the number of eligible women (married and had under-five child) in randomly selected kebeles. After getting the list of eligible women through census, the total sample size was allocated proportionally to the size of the selected kebeles. Then the women were selected by lottery method.

### Data collection procedure

The quantitative data were collected using structured, pre-tested, and interviewer guided questionnaire adapted from similar studies [5,6,11,14-16]. The interview was conducted in the study participants’ usual place of residence*.* The questionnaire was first prepared in English and it was contextualized to suit to the research objective, local situations and language. The quality of the data was assured by translation to Afan Oromo & retranslation to English, pre-testing of the questionnaire, training of the data collectors & supervisors, and close supervision of the data collection processes.

The Afan Oromo version (local language) of the instrument was used to collect the quantitative data. The data were collected by community health agents (CHA) who were intensively trained for two days on the questionnaire and general approaches to data collection.

### Measurements

Women’s autonomy was measured by the composite index of the three constructs of women’s autonomy: control over finance, decision-making power and extent of freedom of movement [5,6,10,16]. A composite measure for each construct was created using the sum of equal weighted binary (1 = responses contributed for higher degree of autonomy versus 0 = otherwise) and three input variables (2 = for women who were able to decide independently, 1 = for joint decision and 0 = otherwise). Based on these values the overall score is found to be 27. Therefore, those women who scored half of the total score i.e. 13.5 and above were considered as highly autonomous while those who scored less than 13.5 were less autonomous.

The index for decision-making power was composed of nine questions. The women were asked “who in her family usually has the final say on the following decisions”: 1. Health care for yourself, 2. Health care for your child, 3. Visit family or relative, 4. Number of children, 5. Use of maternal and child health (MCH) services such as contraception, antenatal care (ANC), preference of delivery site, and child immunization. The possible responses for each item was respondent alone, respondent and husband/partner jointly, respondent and someone else, husband/partner alone & someone else. For each items the response was scored as: 2 if a woman made sole decision, 1 if she was involved with someone [husband/partner or someone else] and 0 otherwise; the sum of the scores were made to represent an overall index of a woman’s decision-making power as indicated by different studies [15-17]. The total score on decision making power was 18. Hence, those women who scored nine and above were categorized as high decision making power whereas those scored less than nine were categorized as women with low decision making power.

The index for control over finance was composed of four items: whether the woman had regular access to a source of money (including both wages earned and gifts or support from family) and whether she stated that she could spend this money without consulting anyone, who decides how the money she earned and her husband’s earnings were used. A score to each of the factors was given as that of index of decision-making power responses, except that 1 and 0 for items with binary responses (i.e. yes or no response). The total score on control over finance was 6. Those women with a score of three and above were considered as having high control over finance, while those women who scored less than three had low control over finance.

The index of freedom of movement consisted of three items pertaining to the woman's ability to leave the house without the company of another adult: whether she could go out to take a child to health facility, to visit family or relative and go to health facility for her own health care. These items were with binary responses (yes or no). Hence, those with ‘yes’ response scored 1 while those with ‘no’ response scored 0. The total score on freedom of movement was 3. Those women who scored one & half and above were considered as high freedom of movement whereas those who scored less than one and half were categorized as low freedom of movement.

Knowledge of women on MCH was assessed by considering knowledge regarding the components of maternal and child health care mainly that addresses ANC, delivery, child immunization services and key danger signs during labor and childbirth. The desired answer were coded as 1; otherwise 0. The total knowledge score was 32. Hence, those mothers who scored above 84% (≥27) were knowledgeable, 50-84% (16–26) moderately knowledgeable and less than 50% (<16) less knowledgeable.

To assess mother’s attitude towards MCH service: eleven items which were positively stated on attitude towards MCH were developed with possible response of agree, undecided and disagree. The measurement scales were given for each item: 3 for agree, 2 for undecided and 1 for disagree. The total attitude score was computed and this value was used for subsequent analysis.

### Operational definitions

Women’s autonomy: is a reflection of women's degree of freedom, relative to men, regarding control over financial resources (economic autonomy); freedom of movement (physical autonomy); opportunity to participate in decisions (decision-making autonomy) about maternal and child health care utilization.

Decision making power: the ability of women to make decision on what to do for their own and children’s health care need.

Freedom of movement: the women’s ability to move to health care facility without seeking permission from other adult (husband’s/partner or someone else) for their own and children’s health care.

Control over financial resources: women’s access to sources of money (her own earning, husband’s/partner’s earning and other sources) and ability to spend it without consulting anyone for their own and children’s health care concern.

### Data processing and statistical analysis

The data were checked for completeness and inconsistencies, edited, coded and entered into SPSS for window version 16.0 (SPSS Inc. version 16.1, Chicago, Illinois). Descriptive statistics, bivariate and multiple logistic regression analyses were carried out. All explanatory variables that showed statistically significant association in the bivariate logistic regression analysis with the outcome variable (women’s autonomy) were entered to the multiple logistic regression model. Hence, the variables entered to the multiple logistic regression analysis were age of the mother, residence, household monthly income, women’s educational status, women’s employment status, family structure, marriage type, husband’s educational status, husband’s employment status, women’s knowledge on MCH and women’s attitude towards MCH. All variables were entered into the model adjusting one another. All tests were two sided and statistical significance level was declared at p-value <0.05.

A letter of ethical approval was obtained from Institutional Review Board (IRB) of Jimma University. Letters of cooperation were obtained from Goba district and Goba town administrative health offices. All women were informed about the purpose of the study and oral informed consent was sought before interview. Additionally, the women were informed about the potential risk and benefits of participating in the study including the right to withdraw from the study at anytime they want. The women were assured about the confidentiality of the information they provided.

## Results

### Socio-demographic characteristics

Of the total 759 eligible women, 706 completed the questionnaire making the response rate 93.1%. The average (±SD) family size for the study population was 5.5(±2.1). The mean (±SD) age of women was 28.3(±5.9) years while the mean (±SD) age of children was 22.9(±15.6) months. Eighty-six percent (86.7%) of women were living in rural areas. Eighty-four percent (84.8%) of women were Oromo by ethnicity. Eighty-one percent (80.9%) of women reported that their husbands have attended formal education. Most (85.7%) of the women were living in nuclear family. Ninety-four percent (94.5%) of the women were living in monogamous type of marriage (Table 1).

**Table 1:** Socio-demographic characteristics and reproductive history of mothers (n = 706) in Goba district, Bale Zone, Southeast Ethiopia, 2011

**Socio-demographic characteristics**	**Category**	**Frequency**	**Percent**
**Age of mother (in year)**	18-19	16	2.3
	20-24	167	23.6
	25-29	249	35.2
	30-34	155	22.0
	35-39	88	12.5
	≥40	31	4.4
**Residence**	Urban	94	13.3
	Rural	612	86.7
**Average monthly income (ETB)**	≤ 500	406	57.5
	501-1000	243	34.4
	≥ 1001	57	8.1
**Ethnicity**	Oromo	599	84.8
	Amhara	101	14.3
	Others^*^	6	0.8
**Religion**	Muslim	394	55.8
	Orthodox	307	43.5
	Protestant	5	0.7
**Mother’s Educational Status**	No education	194	27.5
	Read & write	14	2.0
	Primary (1–8)	390	55.2
	Secondary & above (9+)	108	15.3
**Mother’s Employment Status**	Employed	26	3.7
	Not employed	680	96.3
**Payment status**	Paid	166	23.5
	Not paid	540	76.5
**Family Structure**	Nuclear	605	85.7
	Extended	101	14.3
**Marriage Type**	Monogamous	667	94.5
	Polygamous	39	5.5
**Husband’s Educational Status**	No formal education	134	19.0
	Primary (1–8)	398	56.4
	Secondary (9–12)	140	19.8
	Tertiary	34	4.8
**Husband’s Employment status**	Employed	48	6.8
	Not employed	658	93.2
**Number of children born alive**	1-2	269	38.1
	3-4	222	31.4
	≥5	215	30.5
**Number of children alive now**	1-2	282	39.9
	3-4	245	34.7
	≥5	179	25.4
**Age of children**	<1 years	206	29.2
	1-5years	500	70.8

*includes: Tigrie, Guragie & Wolayita.

### Dimensions of women’s autonomy

**Figure 1:**
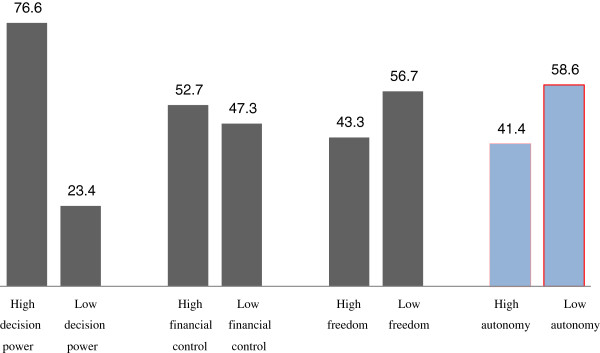
Women’s autonomy measured through decision making power, freedom of movement and control over finance in Goba district, Bale Zone, Southeast Ethiopia, 2011.

Sixty-five percent (65.2%) of women had regular access to a source of money, of which 38.1% were able to use the money by their independent decision. In this study, 49.6% of women were autonomous to take their child to health facilities while 43.9% of women were free to go to health facility for their own health care service consumption. The mean (±SD) score of women on decision making power index was 8.9(±3.2). Concerning women’s freedom of movement, 56.7% of women had lower freedom of movement. The mean (±SD) score of women on control over financial resource was 2.6(±1.2) and 52.7% of women had higher financial control. The overall women’s autonomy mean (±SD) score was 12.6 (±4.6) indicating that 58.6% of the women had lower autonomy while the remaining 41.4% had higher autonomy (Figure 1).

### Factors associated with women’s autonomy

The bivariate logistic regression model showed that women in the age group of 35–39 years were about 4 times more likely to have higher autonomy compared to those women in the age of <20 years [crude odds ratio (COR):3.6 (95% C.I: 1.03-12.04)]. Women residing in urban areas were about 2 times more likely to have higher autonomy than their counterparts in rural areas [COR: 1.7(95% C.I: 1.12-2.69)]. Women’s education was statistically significant with their autonomy. Those women who have attended primary education [COR: 1.8 (95% C.I: 1.27-2.62)], and secondary and above (9+) education [COR: 3.9 (95% C.I: 2.38-6.32)] were about 2 and 4 times more likely to have higher autonomy as compared to those women with no formal education, respectively. Those women who did not earn their own income were less autonomous (Table 2).

**Table 2:** Bivariate and multiple logistic regression model for factors associated with women’s autonomy at house-hold level in Goba district, Bale Zone, Southeast Ethiopia, 2011

**Variables**	**Women’s autonomy**		**Higher autonomy**	
	**Lower autonomy (%)**	**Higher autonomy (%)**	**COR[95%CI]**	**AOR[95%CI]**
Age of mother (year)				
<20	12(75.0)	4(25.0)	1.0	1.0
20-24	107(64.1)	60(35.9)	1.68[0.52,5.45]	0.92[0.26,3.30]
25-29	155(62.2)	94(37.8)	1.82[0.57,5.81]	0.80 [0.23,2.82]
30-34	84(54.2)	71(45.8)	2.54[0.78,8.21]	1.20 [0.33,4.29]
35-39	40(45.5)	48(54.5)	3.60[1.03,12.04]*	1.44 [0.39,5.38]
≥40	16(51.6)	15(48.4)	2.81[0.74,10.67]	1.64 [0.37,7.18]
Residence				
Urban	44(46.8)	50(53.2)	1.74[1.12,2.69]*	0.85 [0.44,1.65]
Rural	370(60.5)	242(39.5)	1.0	1.0
Monthly income (ETB)				
≤ 500	289(71.2)	117(28.8)	1.0	1.0
501-1000	109(44.9)	134(55.1)	3.04[2.18,4.23]*	2.07[1.37,3.13]*
≥ 1001	16(28.1)	41(71.9)	6.33[3.42,11.72]*	3.32[1.62,6.78]*
Ethnicity				
Oromo	358(59.8)	241(40.2)	1.0	
Amhara	54(53.5)	47(46.5)	1.29[0.85,1.98]	
Others	2(33.3)	4(66.7)	2.97[0.54,16.35]	
Religion				
Muslim	218(55.3)	176(44.7)	0.20[0.22,1.82]	
Orthodox	195(63.5)	112(36.5)	0.14[0.02,1.30]	
Protestant	1(20.0)	4(80.0)	1.0	
Mother’s educational status				
No formal education	148(71.2)	60(28.8)	1.0	1.0
Primary (1–8)	224(57.4)	166(42.6)	1.83[1.27,2.62] *	1.22[0.80,1.88]
Secondary & above (9+)	42(38.9)	66(61.1)	3.88[2.38,6.32] *	1.28 [0.66,2.48]
Mother’s employment status				
Employed	6(23.1)	20(76.9)	5.00[1.98,12.61]*	1.85[0.61,5.55]
Not employed	408(60.0)	272(40.0)	1.0	1.0
Payment type				
Paid	91(54.8)	75(45.2)	1.23[0.86,1.74]	
Not paid	323(59.8)	217(40.2)	1.0	
Family structure				
Nuclear	366(60.5)	239(39.5)	0.59[0.39,0.90]*	0.53 [0.33,0.87]*
Extended	48(47.5)	53(52.5)	1.0	1.0
Marriage type				
Monogamous	384(57.6)	283(42.4)	2.46[1.15,5.26]*	3.18 [1.35,7.50]*
Polygamous	30(76.9)	9(23.1)	1.0	1.0
Husband’s educational Status				
No formal education	91(67.9)	43(32.1)	1.0	1.0
Primary (1–8)	245(61.6)	153(38.4)	1.32[0.87, 2.00]	0.85 [0.53,1.39]
Secondary (9–12)	68(48.6)	72(51.4)	2.24[1.37,3.66]*	1.17 [0.64,2.13]
Tertiary (12+)	10(29.4)	24(70.6)	5.08 [2.23,11.56]*	0.61 [0.16,2.29]
Husband’s employment status				
Employed	11(22.9)	37(77.1)	5.32[2.66,10.61]*	3.75 [1.24,11.32]*
Not employed	403(61.2)	255(38.8)	1.0	1.0
Mother’s knowledge on MCH				
Knowledgeable	31(28.7)	77(71.3)	10.25[6.08,17.28]*	6.28[3.46,11.43]*
Moderately knowledgeable	185(52.6)	167(47.4)	3.72[2.55,5.44]*	2.95[1.94,4.48]*
Not knowledgeable	198(80.5)	48(19.5)	1.0	1.0
Mother attitude towards MCH			1.18[1.12,1.25]*	1.12[1.05,1.20]*

*Statistically significant at P < 0.05 COR: crude odds ratio AOR: adjusted odds ratio.

Women who were in a monogamous marriage were 2.5 times more likely to be highly autonomous as compared to women engaged in a polygamous marriage [COR: 2.5(95% C.I: 1.15-5.26)]. Meanwhile, those women living in a nuclear family were 41% less likely to be highly autonomous as compared to those women in an extended family [COR:0.59(95% C.I: 0.39-0.90)] (Table 2).

Husband’s with secondary educational status [COR: 2.2(95% C.I: 1.37-3.66)] and husband’s employment status (being employed) [COR: 5.3(95% C.I: 2.66-10.61) were significantly associated with women’s autonomy to seek health care services for themselves and their children. Women who were knowledgeable about maternal and child care services were10 times more likely to be highly autonomous as compared to those who were not knowledgeable [COR: 10.3 (95% C.I: (6.08-17.28)] (Table 2).

In the multiple logistic regression model, after adjusting for the potential confounders; household monthly income, husband’s employment status, family structure, type of marriage, knowledge and attitude of women towards maternal and child health care services were the final predictors of women’s autonomy. In this study, those women who were living in a nuclear family had lower odds of autonomy as compared to those women who were living in an extended family [AOR: 0.53 (95% C.I: 0.33-0.87)]. Women who were in a monogamous marriage had a higher odds of increased autonomy as compared to women engaged in a polygamous marriage [AOR: 3.2 (95% C.I: 1.35-7.50)]. Women who had employed husband had higher odds of increased autonomy as compared to women who had an unemployed husband [AOR: 3.7 (95% C.I: 1.24-11.32)] (Table 2).

Those women who were knowledgeable [AOR: 6.3 (95%C.I: 3.46-11.43)] and moderately knowledgeable [AOR: 3.0 (95% C.I: 1.94-4.48)] about maternal and child health services respectively were 6 and 3 times higher odds of increased autonomy as compared to their counterparts. Similarly, women who had a favorable attitude score had higher odds of autonomy as compared to those women with a lower attitude score towards MCH [AOR:1.12(95% C.I: 1.05-1.20)] (Table 2).

## Discussion

In Ethiopia health services utilizations are very low, particularly maternal and child health care. Women’s autonomy regarding maternal and child health are important factors in determining health seeking behavior. The current study used validated instruments and measured women’s autonomy and identified factors associated with it. Such studies will improve maternal and child health service utilizations. Additionally, these studies can serve as a baseline data against which future progress regarding women’s autonomy can be measured.

In this study, 65.2% of women have access to money and out of those women who have access to money; only 38.1% of them were autonomous to use the money for health care service consumption without consulting others. Our results here are comparable with a report from North India which indicated that 59% of women had unrestricted access to money while 55% of women spent money without consulting other person [6]. In our study, half (49.6%) of women were able to take their children to a health facility, which is lower than a report from North India, where 84% of women were able to take their child to a health facility without requesting permission [6]. This difference might be due to socio-economic, cultural and gender role differences between women in Ethiopia and North India.

In our study, household monthly income was positively associated with women’s autonomy. Those women with higher household monthly income were more likely to be highly autonomous as compared to their counter parts. This finding is consistent with the Ethiopian national level study result that revealed women in the highest wealth quintile were highly decisive on health care utilization for their own/child health care services utilization [14]. Similar finding was also reported in Nepal [15].

This study revealed that women who had an employed husband were more likely to be highly autonomous as compared to women who had an unemployed husband. A study from Nepal similarly found that the husband’s occupation was associated with women’s autonomy [18]. This might be due to the cumulative effect of income on the economic status of the family in general and the women in particular, that create better conditions for the women to be autonomous.

Women who were in monogamous marriage were more likely to be autonomous. This could be due to the fact that men who have two or more wives at a time would not be able to satisfy their financial needs. On the other hand most women who have engaged in polygamy might be rural, economically deprived/less earnings and uneducated women.

Women in nuclear family were less likely to be autonomous. Contrarily, evidence from Pakistan showed that women in nuclear households were more likely to be autonomous in participating and passing decisions at family level [19,20]. This might be due to the nature of the people added to the family i.e. they might be related to the mother in any case they support her in household decision making process. Mostly, if women live with their mother-in-law, their autonomy may be negatively affected. As evidenced by a study from India, women who were not living with their mothers-in-law had a higher involvement in the decision-making process [6].

In our analysis, knowledge and attitude towards maternal and child health care services were associated with increased women’s autonomy. This might be due to the fact that those women who have knowledge and a favorable attitude towards maternal and child health care services could easily influence their husbands and significant others.

Although women’s autonomy is a complex measure and there is no commonly agreed single definition for it, this study tried to address the most frequently used dimension of autonomy measures by different scholars [5,6,10,16]. This might be considered as the strength of the study. However, the study has limitations as mother might not recall her experience of visiting health care services autonomously for their own and their child health problems. As a result women’s autonomy is subject to potential recall bias. This study was also unable to assess women’s autonomy to specific maternal and child health services (such as: ANC, Delivery services, Immunizations etc.) by which women’s autonomy might not be interpretive to specific services. Finally, this study used a cross-sectional study design and hence it is not possible to establish causal association between dependent and independent variables.

## Conclusion

This study showed that nearly half of the women were found to have lower control over financial resources. More than half of women were found to have lower autonomy. Household monthly income, having an employed husband, being in an extended family structure, being in a monogamous marriage, being knowledgeable and having a favorable attitude score towards maternal and child health care services were positively associated with women’s autonomy. Therefore, these factors should be taken into account while designing interventions. In addition, targeted, community oriented and promotive strategies including women empowerment through involving women and men in income generating means such as small and microenterprises are recommended. Furthermore, strengthening the health extension workers (HEWs) activities that are directed in increasing awareness and improving attitude towards maternal and child health care services are recommended to empower women to seek these services.

## Competing interests

All authors declare that they have no competing interests.

## Authors’ contributions

DN conceived and designed the study, participated in the process of data collection on the field, performed the data analysis, interpreted the data, and drafted the manuscript and critically reviewed it. AG, MA, TS and KD assisted in designing, analysis, interpretation, manuscript preparation and reviewed the manuscript critically. All authors read and approved the final manuscript.

## Pre-publication history

The pre-publication history for this paper can be accessed here:

http://www.biomedcentral.com/1472-6874/14/79/prepub
